# The Role of Matrix Protein 2 Ectodomain in the Development of Universal Influenza Vaccines

**DOI:** 10.1093/infdis/jiz003

**Published:** 2019-04-08

**Authors:** Xavier Saelens

**Affiliations:** 1 VIB-UGent Center for Medical Biotechnology, Ghent; 2 Department of Biomedical Molecular Biology, Ghent University, Belgium; 3 Department of Biochemistry and Microbiology, Ghent University, Belgium

**Keywords:** M2e, universal influenza vaccine, clinical studies, Fc receptors

## Abstract

The influenza A virus matrix protein 2 ectodomain (M2e) is a universal influenza A vaccine candidate. Numerous studies in laboratory mice, but very few in natural influenza A virus hosts, have demonstrated that M2e-based vaccines can provide protection against any influenza A virus challenge. M2e-based immunity is largely accomplished by IgG and early stage clinical studies have demonstrated that the vaccine is safe. Yet M2e is considered a difficult target to develop as a vaccine: it does not offer sterilizing immunity and its mode of action relies on Fcγ receptor-mediated effector mechanisms, most likely in concert with alveolar macrophages. In a human challenge study with an H3N2 virus, treatment with a monoclonal M2e-specific human IgG was associated with a faster recovery compared to placebo treatment. If the universal influenza vaccine field incorporates this antigen into next generation vaccines, M2e could prove its merit when the next influenza pandemic strikes.

The influenza A virus matrix protein 2 (M2) is essential for virus propagation. M2 was discovered by Robert Lamb, and it is now known that the protein fulfills at least 3 critical functions [1, 2]. First, its ion channel activity is required for the disassembly of the viral core. This process starts in the acidic environment of the endosomes, shortly after virion uptake by the infected cells. Initially, at a pH of 6.5–6.0, the M2 channel enables the flux of protons into the virion interior, which weakens the interactions between matrix protein 1 (M1) and the viral ribonucleoproteins (vRNPs) within the viral core. In the more acidic (pH 5.4–6.0) late endosomes, M2 also starts to conduct potassium ions into the virion, which results in the disruption of the vRNP-vRNP interactions [2, 3]. The ensuing pH-triggered hemagglutinin (HA)–mediated membrane fusion finally releases the “primed” vRNPs into the cytosol, ready to migrate to the nucleus where the viral transcription and replication can start.

Second, M2 is required for virus assembly and budding. The membrane distal end of the cytoplasmic part of M2 interacts with M1, which leads to virion assembly [4]. The membrane proximal, cytoplasmic, amphipathic α-helix of M2 controls the budding process. In the plasma membrane of the infected cell, M2 accumulates at the rim of HA- and neuraminidase-containing lipid rafts, alters membrane curvature. and eventually pinches off newly assembled virions [5]. Finally, M2 perturbs several host cell functions. In myeloid cells, its ion channel function may activate inflammasomes, whereas a conserved LC3-interacting motif close to the carboxyterminal end of its cytoplasmic domain interferes with autophagy (Figure 1A) [6, 7]. The functional orthologue of M2 in influenza B virus is named *BM2*. Like M2, BM2 can shuttle protons across membranes and it interacts with M1 of influenza B virus [8]. The sequences of M2 and BM2 are very different.

**Figure 1.**
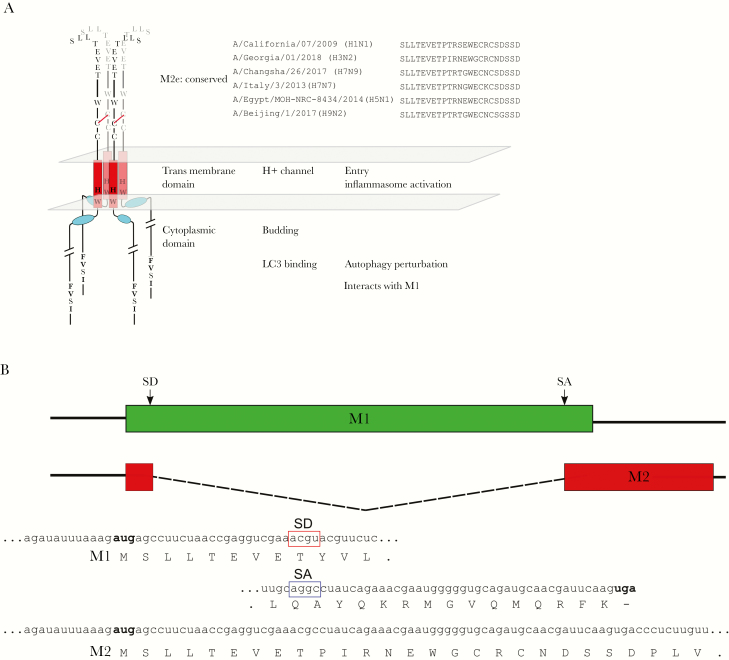
Schematic representation of matrix protein 2 (M2). *A,* M2 is depicted as a tetrameric transmembrane protein composed of 2 dimers that are covalently linked by a disulfide bond between pairs of cysteines (*red line*). The M2 ectodomain (M2e), at the N-terminus, faces the extracellular side of the lipid bilayer that is represented by 2 transparent gray parallelograms. The sequence conservation of M2e is illustrated by the listed sequences from diverse influenza A viruses (all from cases in humans). The red rectangles represent the transmembrane domain. The histidine and tryptophane residues at positions 37 and 41 of M2, respectively, are essential for the proton-selective ion channel activity and are shown in the single letter code. The blue ovals represent the α-helix required for virion budding. These helices are oriented nearly parallel with the cytoplasmic membrane. Near the C-terminus of the cytoplasmic domain of M2, a conserved FVXI motif is shown that is important for LC3 binding. The C-terminal part of M2 also interacts with matrix protein 1 (M1). *B,* Schematic diagram of the M1 and M2 open reading frames, illustrating the overlap between M2e and M1. The nucleotide and amino acid sequences represent the start and end of M1 and the start of M2 and are derived from A/Hong Kong/01/1968(H3N2). Abbreviations: SA, splice acceptor; SD, splice donor.

## THE M2 ECTODOMAIN: CONSERVED, POORLY-STRUCTURED, AND YET IMMUNOPROTECTIVE

The aminoterminal part of M2 that protrudes from the membrane is the M2 ectodomain (M2e) and is 23 amino acid residues long. Its strong sequence conservation across all influenza A virus subtypes may suggest that M2e fulfills an important role in the virus life cycle, yet we know surprisingly little about its function. Presumably, M2e merely plays a role in controlling the N-out and C-in orientation of M2, a type III membrane protein, although there is no particular primary sequence requirement known for such a role, apart from a preference for an acidic residue that precedes the transmembrane region [9, 10]. The part of genome segment 7 that codes for the first 9 amino acid residues of M2 is packed with information, which explains its very high sequence conservation. It comprises a packaging signal for viral RNA segment 7, the beginning of the open reading frame of 2 different proteins (M1 and M2), as well as the splice donor for the M2 transcript [11]. The relative sequence conservation of amino acid residues 10–24 of M2e is dictated by the overlapping C-terminus of M1. This overlap is in a different reading frame from that of M1 and also comprises the splice acceptor for the M2 messenger RNA (Figure 1B).

The first 9 amino acid residues of M1 (identical in M2) fold into an α-helix, which is part of a helical bundle structure that is conserved in orthomyxoviruses [12, 13]. Solid-state magnetic resonance analysis of M2 embedded in artificial lipid bilayers suggests that M2e can adopt a β-strand or, in the presence of cholesterol, an α-helical conformation [14]. An M2e peptide complexed with an antibody-derived fragment antigen-binding (Fab) fragment, on the other hand, can fold in at least 2 very different conformations dependent on the specificity of the monoclonal antibodies used [15, 16]. The interactions of the M2e residues with the antibody paratope revealed by these M2e peptide–Fab complexes explain why some influenza A viruses, which have a slightly different M2e sequence, could resist recognition by a particular monoclonal antibody. This suggests that the M2e peptide–Fab complexes are biologically relevant. It would be interesting to use modern, Å-resolution quality cryoelectron microscopy to reveal how M2e-specific monoclonal antibodies bind their target in the context of a lipid membrane and to clarify, for example, whether any interaction of the antibody variable domains with the lipid membrane is implicated. Are the M2e moieties within a single M2 tetramer accessible for >1 Fab? Or would M2e-specific single-domain antibodies be needed to accomplish this? No M2e-specific antibodies that recognize quaternary surfaces on tetrameric proteins have yet been isolated.

Since the first report that a mouse immunoglobulin (Ig) G1 monoclonal antibody directed against M2e, and the finding that active vaccination of mice with a full-length recombinant M2 preparation or, more elegantly, with recombinant M2e-displaying virus-like particles, could protect mice against a potentially lethal influenza A virus challenge, hundreds of publications have confirmed the immunoprotective potency of M2e-based vaccines [17–19] (reviewed in 20). The main conclusions of these studies are that IgG antibodies specific for M2e are essential for the protection and that M2e-based immunity can reduce virus replication and disease associated with influenza A virus infection, and, importantly, can protect against challenge with any influenza A virus subtype. The extracellular part of BM2 is only 7 amino acid residues long, most likely too short to induce a meaningful antibody response.

Some M2e-fusion constructs provide better protection than others. In general, recombinant nonenveloped viruslike particles (VLPs) as a carrier for M2e antigens are the preferred vaccine formats to elicit M2e-specific immune responses. Examples include the use of hepatitis B core and bacteriophage Qβ capsids, which have the advantage that they can be produced in prokaryotic expression systems using culture media that are free of animal-derived products [19, 21]. In addition, these VLPs can package immunostimulatory nucleic acids that induce T-helper 1–biased adjuvant effects [21, 22]. Vaccination with M2e VLPs also results in a (strong) immune response directed against the carrier capsids, which has been considered acceptable by regulatory authorities for early-stage clinical testing of M2e vaccine candidates. However, the rollout of hepatitis B core-based VLPs to induce an immune response against a grafted antigen such as M2e could, in the longer run, confound the interpretation of serological diagnostic tests performed to assess a patient’s hepatitis B carrier status.

VLPs with multiple head-to-tail copies of M2e induce much stronger M2e-specific immune responses compared with single M2e fusion constructs, presumably because the antigen is better accessible for the B-cell receptor, and an avidity effect is created. This way, different M2e variants can also be incorporated into the same vaccine construct, which results in better coverage of the limited M2e diversity (M2e of human H3N2 and H1N1 viruses that have circulated since 2009, for example, differs at 4 positions). Such M2e repeats have been successfully fused with a tetramerizing leucine zipper followed by a transmembrane domain and produced as enveloped, insect cell–derived VLPs [23]. The use of decameric human respiratory syncytial virus nucleoprotein-based nanorings as carriers of tandem repeat copies of M2e has also been explored. These nanorings can be produced in *Escherichia coli* and induce robust protective anti–M2e IgG and IgA responses after intranasal immunization of mice [24].

In general, genetic or vectored full-length M2 vaccine constructs induce a weak M2e-specific antibody response, which is in line with the naturally low humoral immune response against M2. Still, heterologous prime-boost schedules with M2 expressing plasmid DNA, followed by an adenoviral M2 expression vector, can induce fairly strong anti-M2e antibody responses and protection that is in part mediated by T cells [25]. Heterologous prime boosts are more difficult to bring into practice than a single-shot or homologous booster vaccine regimen. However, the adult population seems to be already primed with M2, as evidenced by the presence of serum IgG that is reactive against M2 expressed by 293FT cells. Moreover, there is evidence that M2-specific antibodies were boosted on infection with the H1N1 2009 pandemic virus [26]. Therefore, a single immunization with a vectored M2 expression vector or, preferentially, a recombinant M2e-displaying VLP, may effectively boost the M2(e)-specific responses in adults, including elderly adults, but may not work as well in unprimed children.

## M2E: A SIMPLE ANTIGEN THAT PROTECTS IN A COMPLEX WAY

Few influenza A viruses are susceptible to direct in vitro growth inhibition by M2e-specific IgG. Interestingly, the pandemic 1957 H2N2, 1968 Hong Kong, and H1N1 USSR 1977 viruses belong to these exceptions and show a reduced plaque phenotype in the presence of 5 μg/mL of the M2e-specific monoclonal antibody 14C2 [27]. On the other hand, M2e-immunized mice are protected against challenge with any influenza A virus that has been tested so far, as long as the severity of the challenge is relatively low (1–4 times the medial lethal dose). This discrepancy between the in vitro and in vivo antiviral effects of M2e-based vaccines is one reason why M2e is sometimes considered a “difficult target”: there is no easy way to set up an in vitro antiviral activity assay that recapitulates what probably happens in an M2e-immunized host. Such an assay is important because it could be standardized and used to predict the likelihood that the recipient of an M2e-based vaccine will be protected from disease, severe disease, or hospitalization due to influenza A.

**Figure 2.**
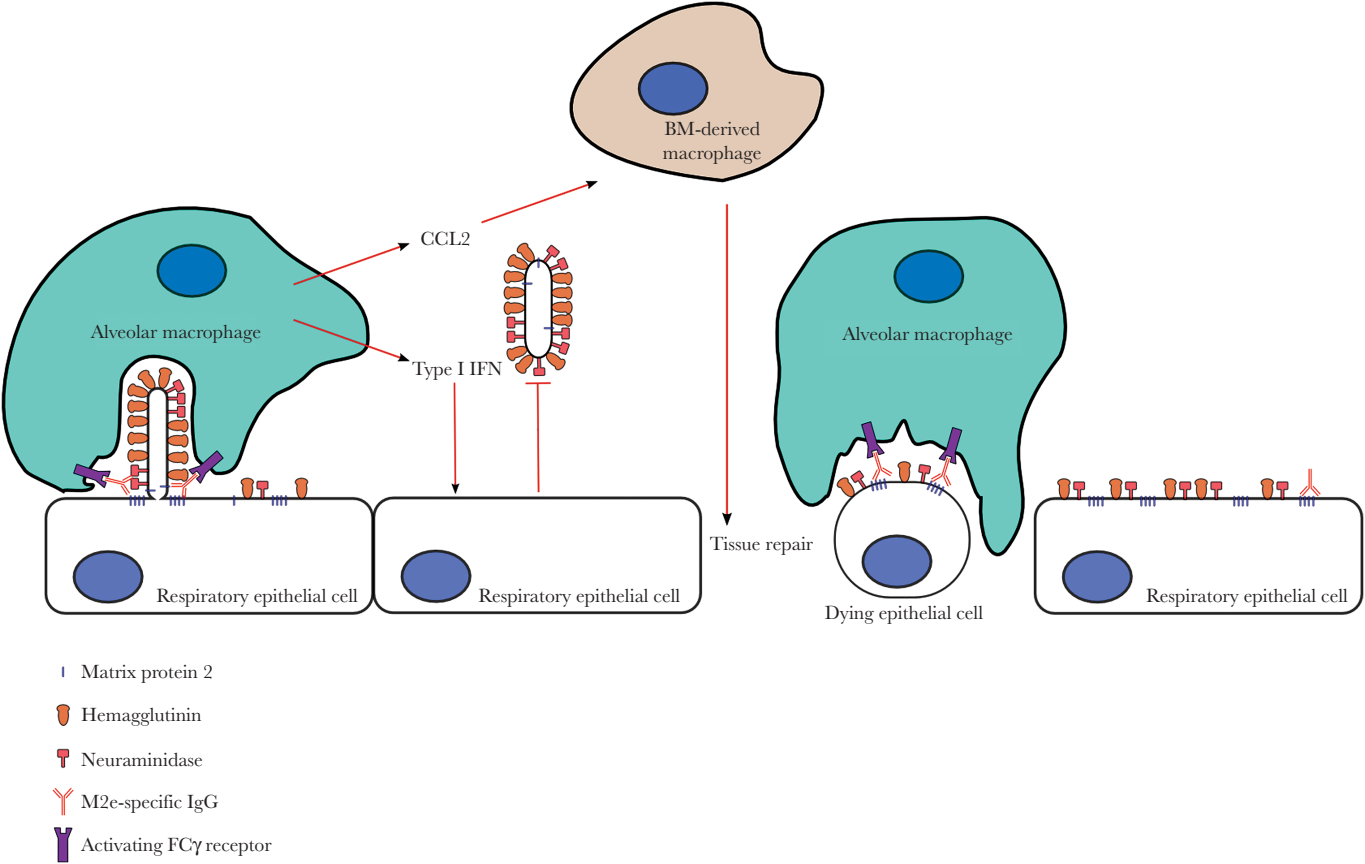
Schematic representation of the likely mechanism of protection of matrix protein 2 (M2) ectodomain (M2e)–specific immunoglobulin (Ig) G. Airway epithelial cells infected with influenza A virus display hemagglutinin, neuraminidase, and M2 on their surface and bud off newly produced virions. M2 at the neck of the budding virion can be opsonized with anti–M2e IgG, which in turn is bound by activating Fcγ receptors on alveolar macrophages. This way, macrophages can take up budding virions and M2-containing membrane fractions from an infected respiratory epithelial cell. Infected cells that detach from neighboring cells can be opsonized by anti–M2e IgG and become phagocytosed by alveolar macrophages in an Fcγ receptor–dependent way. Activated macrophages can also produce type I interferon (IFN), which has antiviral activity by inducing an antiviral state in the epithelial cells. In addition, type I IFN can up-regulate the chemokine CCL2, which attracts bone marrow–derived macrophages that promote tissue repair.

Thanks to mouse genetics, antibody Fc engineering, and in vivo cell depletion studies, the mechanism of protection by M2e-specific IgG is now well established. The consensus view is that IgG antibodies form immune complexes on M2-expressing infected respiratory epithelial cells, which are recognized by activating Fcγ receptors expressed on alveolar macrophages (Figure 2) [28–30]. Interestingly, alveolar macrophages are also essential for the protection by broadly reactive HA-specific monoclonal antibodies, presumably by performing antibody-dependent cellular phagocytosis of influenza virions and infected cells [31]. Given that there are very few M2 molecules present in influenza virions, it is likely that infected cells rather than virions are the target of antibody-dependent phagocytosis by alveolar macrophages. Elimination by alveolar macrophages of infected cells, and possibly of virions that are in the process of budding from the infected cells (macrophages prefer to engulf rigid particles [32]), would slow down the virus replication-induced disease process.

Alveolar macrophages are also an important source of type I interferon (IFN) in response to respiratory viral infections [33, 34]. Type I IFN produced by alveolar macrophages can elicit a direct antiviral response in neighboring cells. In addition, type I IFN induces expression of the chemokine CCL2, which recruits inflammatory monocytes that in turn can promote epithelial cell repair [35]. Interestingly, there is evidence that M2e-based immune protection may even stimulate an adaptive immune response against other influenza proteins. First, nucleoprotein-specific CD8^+^ T-cell responses are comparable in saline- and M2e VLP–vaccinated mice after a sublethal influenza A virus challenge, even though the M2e-immune animals were very well protected, presenting with minimal loss of body weight [36, 37]. Possibly, anti–M2e IgG immune complexes are also recognized by dendritic cells, which thereby can take up, process, and present virus-containing cell fractions to T cells in the draining lymph nodes. T-cell responses directed against the conserved internal influenza gene products are important because they are associated with broad protection [38].

Second, M2e-specific CD4^+^ T cells can contribute to protection in their own right, but they also stimulate HA-specific antibodies on challenge virus infection [39]. So here is a vaccine candidate that is easy to produce, can provide very broad protection against influenza A virus challenge, and does not stop the host from mounting an immune response to other viral antigens on virus encounter. Why is this product not yet licensed? To answer that question, it is important to scrutinize M2e vaccination studies in hosts that are naturally susceptible to influenza and consider what has been learned from phase I clinical studies.

## M2E-BASED VACCINES IN HUMANS

There is some evidence that an M2e-based vaccine approach can suppress influenza A virus replication in ferrets. These carnivores are considered relevant for human influenza, given their susceptibility to human influenza virus isolates, the clinical features they display on influenza virus infection, and the distribution of the virus receptors in their respiratory tract, which is similar to that in humans [40]. Ferrets that were immunized with a vaccine comprising M2e linked to 2 different carriers mounted robust M2e-specific serum IgG titers and showed reduced virus shedding in the lungs after challenge with PR8 virus [41]. A 2017 study found that immunization of ferrets with bacteria-derived outer membrane vesicles displaying 4 tandem repeat copies of M2e reduced in lung virus titers more than a conventional influenza vaccine after challenge with H1N1 2009 pandemic virus [42]. In pigs, modest protection against disease after aerosol challenge with a nebulized swine H1N1 virus (10^8^ median tissue culture infective dose) was reported despite the mismatch of 6 amino acid residues between the vaccine (adjuvanted M2e VLPs) and challenge virus M2e [43]. Finally, M2e-based vaccines can partially protect chickens against experimental challenge with avian influenza viruses, including a highly pathogenic H5N1 strain [44, 45].

The scant data about the possible clinical benefit that anti-M2e responses may provide look promising. First, preexisting humoral immunity to M2 (meaning reactivity of serum IgG with M2-expressing mammalian cells, thus primarily directed against M2e) showed a tendency toward an inverse correlation with the incidence of H1N1 2009 pandemic virus infection by age group in US residents [26]. Second, phase I clinical studies have shown that M2e-based vaccines are safe, although volunteers who had received a low dose of a M2e-flagelin fusion protein, administered subcutaneously, presented with local and systemic adverse effects, most likely due to the Toll-like receptor 5–stimulating flagellin component (clinical trials NCT00819013, NCT00921973, NCT00921947, NCT00921206, NCT00603811, and NCT01184976). Finally, anti–M2e IgG can be protective in humans. This was shown in a placebo-controlled, double-blind, controlled challenge study in humans with the recombinant human IgG1 monoclonal antibody TCN-032, which recognizes the highly conserved N-terminus of M2. Intravenous administration of TCN-032 at 40 mg/kg 1 day after intranasal infection with a human H3N2 virus significantly reduced the total daily symptoms compared with placebo treatment, by 35%, and resulted in 2-day-faster recovery [46]. Importantly, the treatment was also safe.

## FUTURE PERSPECTIVES FOR ANTI-M2E IMMUNE PROTECTION

The earliest patent applications on M2e-based vaccines are expired by now, but research and clinical development of these vaccines is still ongoing. The Research Institute of Influenza in St Petersburg (The Russian Federation) has started a phase I study to assess the safety and immunogenicity of a hepatitis B core–based particle carrying 4 copies of M2e inserted in the immunodominant loop of the hepatitis B core capsomer, and adjuvanted with Derinat (Liudmila Tsybalova, personal communication on May 22, 2018, Technomedservia Pharmaceutical Company, Mironovskaya, Russia) [47]. The Theraclone treatment study showed that 500 µg/mL of a monoclonal IgG1 antibody specific for M2e (assuming a total blood volume of 6 L in a normal healthy adult with an antibody dosing of 40 mg/kg) could bring clinical relief when administered 1 day after experimental virus challenge [46].

It is highly unlikely that an active vaccination protocol could induce such a high peak, let alone sustained, concentration of M2e-specific IgG in circulation. However, considering that only an estimated 0.2% of that monoclonal probably reached the lumen of the lungs, the estimated 10-µg/mL concentration of the TCN-032 antibody at the infection site may actually have done the therapeutic job [48]. Furthermore, prophylaxis is likely to require less antibody to suppress virus replication, perhaps 10-fold less, which would correspond to 1 µg/mL of antibody in the lung lumen. These are levels that may be attainable, at least in theory, with active mucosal immunization. To our knowledge intranasal immunization studies in humans with M2e-based vaccine antigens have not been reported. In addition, an active vaccination strategy would induce a polyclonal immune response, which could have some advantages.

A universal influenza vaccine will very likely have a tremendously beneficial economic and health impact when the next influenza pandemic strikes, provided that such a vaccine is stockpiled and can be distributed rapidly. Alternatively, such a vaccine could be implemented as part of childhood vaccination programs, which would require the induction of long-term immune protection. A disappointment in the phase I clinical study with M2e VLPs was the rapid decline in anti–M2e IgG titers over time, with end-point M2e IgG titers that were hardly above background levels 1 year after the first immunization. A more sustainable immune response may be achieved with the right adjuvant, such as DepoVax (IMV Inc.) or AS01 (GSK) [49, 50]. Finally, for the M2e-based approaches, it is fair to state that broader protection comes with reduced potency, compared with conventional seasonal influenza vaccines. Therefore, an M2e vaccine will probably be developed further as one among multiple components of a broadly protective influenza vaccine. A possible scheme that can be envisioned is to combine M2e with an HA stalk–based vaccine in a prime boost regimen within childhood vaccination programs. Such a combination vaccine could induce broadly reactive anti-influenza immunity and prevent severe disease in the very young.

## Notes

***Acknowledgments.*** Research related to influenza vaccine and antiviral development is supported by Fonds voor Wetenschappelijk Onderzoek (project grant G043515N), by Ghent University Bijzonder Onderzoeksfonds (project BOFGOA2017000502), and by Sanofi Pasteur.

***Potential conflict of interest.*** Research on influenza vaccine development in the author’s laboratory is supported in part by Sanofi Pasteur. The author is also named as an inventor on a patent application that describes an M2e-based antiviral therapy. The author has submitted the ICMJE Form for Disclosure of Potential Conflicts of Interest. Conflicts that the editors consider relevant to the content of the manuscript have been disclosed.
